# Effect of patient education in improving quality of life, fatigue and anxiety in people diagnosed with lung cancer: systematic review

**DOI:** 10.1007/s00520-026-10331-8

**Published:** 2026-02-03

**Authors:** Laura Mª Árbol-Guerrero, David Ortega-Valle, Hermann Fricke-Comellas, Mª Jesús Casuso-Holgado, María Jesús Muñoz-Fernández, Patricia Martínez-Miranda

**Affiliations:** 1 Hospital Universitario Son Espases, Carretera de Valldemossa, 79, 07120 Palma, Islas Baleares, Spain; 2 Department of Physiotherapy, Faculty of Nursing, Physiotherapy and Podiatry, Universidad de Sevilla, C/Avicena S/N, 41009 Seville, Spain; 3 CTS 1110, UMSS Research Group, Andalusia, Seville, Spain; 4 Instituto de Biomedicina de Sevilla-IBiS (Hospitales Universitarios Virgen del Rocío y Macarena / CSIC/ Universidad de Sevilla), Seville, Spain; 5 Department of Physiotherapy, Avd. de los Cipreses S/N, University School Francisco Maldonado, 41640 Osuna, Spain; 6 Department of Clinical and Health Sciences, Universidad Loyola de Andalucía, Seville, Spain

**Keywords:** Cancer, Patient education, Quality of life, Fatigue, Anxiety

## Abstract

**Purpose:**

To assess the effect of patient education on quality of life, fatigue, and anxiety in patients diagnosed with lung cancer.

**Methods:**

An electronic search was conducted across four databases (PubMed, Web of Science, CINAHL, and Scopus) using a combination of terms including *lung neoplasms*, *health education*, *educat* (truncated), *quality of life*, *fatigue*, and *anxiety*. The Cochrane RoB 2 tool and the TIDieR checklist were used to assess risk of bias and intervention replicability, respectively. The GRADE approach was applied to evaluate the certainty of the evidence. Study selection, data extraction, and all assessments were carried out independently by two reviewers. Where appropriate, data were pooled using meta-analysis (95% confidence interval [CI]).

**Results:**

Seventeen studies were included in the qualitative synthesis, and thirteen in the quantitative analysis, comprising a total sample of 1799 participants. The meta-analysis demonstrated that, compared with controls, patient education interventions had a statistically significant and large effect on improving quality of life (SMD = 0.98; 95% CI [0.26, 1.69], p = 0.007, I^2^ = 96%), anxiety (SMD = -1.75; 95% CI [-2.74, -0.77], p = 0.0005, I2 = 98%) and fatigue (SMD = -0.091; 95% CI [-1.61, -0.22], p = 0.01, I2 = 88%). In all cases, heterogeneity remained high. However, the educational content of the interventions was generally consistent, with most being delivered in a face-to-face format.

**Conclusions:**

Patient education appears to be an effective approach for improving quality of life, fatigue, and anxiety in individuals with lung cancer. Nevertheless, these findings should be interpreted with caution, as the certainty of the evidence was rated as very low.

**Supplementary Information:**

The online version contains supplementary material available at 10.1007/s00520-026-10331-8.

## Introduction

Lung cancer is currently the most common cancer worldwide, with almost 2.5 million diagnoses and 1.8 million deaths in 2022. The disease is responsible for nearly one in five cancer deaths (18.7%) [1], with a low global five-year survival rate (6–18%) [2].

Regarding medical treatments, the choice of combinations of therapeutic strategies depends on histological type, tumour location, cancer stage and the patient's level of frailty [3]. Standard treatments usually include surgery, chemotherapy, radiotherapy and molecular targeted therapies. These interventions are often associated with several side effects and sequelae, such as fatigue, anxiety and a significant decrease in quality of life [4]. Currently, different pharmacological and non-pharmacological therapies are used to mitigate these side effects or sequelae.

Patient education stands out as a widely used intervention within the rehabilitation process [5], with the potential to reduce drug use and generate socio-economic benefits [6]. Patient education is thus defined as any intervention led by a healthcare professional with the aim of empowering individuals to manage their health effectively, enhancing autonomy and self-efficacy [7–10]. In oncology, evidence supports the effectiveness of patient education in improving a range of cancer-related outcomes, such as quality of life, pain, fatigue, anxiety, depression, among others [11–17]. On the other hand, in lung cancer specifically, there have been different systematic reviews on the effectiveness of non-pharmacological, self-management and telerehabilitation interventions [18–20] as well as different clinical trials applying education [21–23]. However, until now, no systematic review or meta-analysis had focused specifically on evaluating the effectiveness of education in patients diagnosed with lung cancer, so this study is a pioneer in this field.

This review focuses on quality of life, fatigue and anxiety, which are among the most frequent lung cancer-related symptoms, based on two criteria. Firstly, they are highly prevalent and impact the daily lives of people diagnosed with lung cancer [4]. Secondly, they are frequently included as primary or secondary outcomes in previous clinical trials evaluating educational interventions [21–23]. Thus, this article could be a first approach to the effect of education on lung cancer that could be a precursor to other studies focused on assessing other side effects. Therefore, the aim of this review and meta-analysis is to synthesise the effect of patient education on quality of life (primary outcome), fatigue and anxiety (secondary outcomes) in patients diagnosed with lung cancer.

## Methods

This systematic review was carried out following the PRISMA Declaration recommendations [24]. Its protocol was registered in PROSPERO (registration number CRD42020183757).

The research question was defined following recommendations from the Population, Intervention, Comparison and Outcome measures (PICOS) model as follows: What is the effect of patient education in the multidimensional improvement of quality of life (main variable), anxiety and fatigue (secondary variables) compared with a passive control group or other interventions in adults diagnosed with lung cancer? Inclusion criteria: (1) adults diagnosed with lung cancer in any stage undergoing primary treatment or finalised (survivors) (P); (2) intervention based on the application of patient education in isolation or in combination with another tool (I); (3) compared with a passive control group or a different intervention of education (C); (4) studies that included quality of life, fatigue or anxiety as outcomes (O), randomised clinical trials (RCTs) (S). Exclusion criteria: (1) studies that exclusively evaluated the comparative effectiveness of different patient education modalities, (2) studies that included participants with different types of cancer without reporting a subgroup analysis.

The search was carried out from the databases until 16 April 2025 in four databases (PubMed, Web of Science, CINAHL and Scopus) using the following search terms: 1# Lung Neoplasms, 2# Health Education, 3# educat*, 4# Quality of Life, 5# Fatigue and 6# Anxiety. These terms were obtained using two thesauri (DESC and MESH) and by consulting the keywords used in literature with a similar theme. They were combined to establish the following search strategy: 1# AND (2# OR 3#) AND (4# OR 5# OR 6#). The search strategy was adapted to each database. Filters were used only for study type (clinical trial/randomized clinical trial). There were no other restrictions. In addition, the reference lists of several systematic reviews were scrutinised. A detailed search strategy is shown in Supplementary File 1.

Two independent reviewers (PMM and LMAG) completed the study selection process following the PRISMA model. Another reviewer (HFC) helped when it was necessary. Data extraction was performed by the same reviews following the PICOS model. Information regarding the participants (sample size and gender), intervention (modality and content of education program; time, number and duration of sessions; duration of the entire intervention; professional that developed the program), control group (type of intervention and duration) and outcome variables (quality of life, anxiety and/or fatigue measurement tool, number of measurements, measurement time and obtained results) was extracted.

Two independent reviewers (LMAG and HFC) performed methodological quality assessment using the Cochrane Risk of Bias 2 [25]. The percentage of discrepancy was noted. A third independent reviewer (PMM) evaluated disagreements.

The completeness of intervention descriptions was assessed by one reviewer (HFC) with the Template for Intervention Description and Replication (TIDieR) checklist [26]. This tool evaluates whether interventions were reported in enough detail in primary studies to ensure replicability.

The findings were described narratively and where possible, study results were pooled, and meta-analysis was conducted based on the same underlying outcomes (quality of life, anxiety and fatigue) and the same time point (immediately after intervention). Results of studies with different phases of treatment (undergoing oncologic therapies or survivor phase) were not combined in the same meta-analysis. Three corresponding authors were contacted several times for data requirements, but no answer was provided [23, 27, 28]. As a result, a total of 13 RCTs were included in our meta-analysis. Data were pooled with an inverse variance weighting method, and standard mean differences (SMDs) were estimated using the Hedge’s g method. The sizes of the Hedge’s g effect can be classified into small effect (g = 0.2), medium effect (g = 0.5) or large effect (g = 0.8). Fixed or random effects models were chosen according to the degree of heterogeneity, which we assessed using the I^2^ coefficient. Specifically, for I^2^ > 50%, which indicates substantial heterogeneity, we used random effects models, and when I^2^ < 50%, which indicates substantial homogeneity, we used fixed effect models. Sensitivity analyses were developed to detect outliers or influential cases by an exploratory analysis of the data (leave-one-out method). If one study was detected as an outlier or influential case, it was removed from the meta-analysis. Finally, formal tests for assessing publication bias are not recommended when the number of studies included in each meta-analysis is small [29, 30]. Although we developed funnel plots for asymmetry inspection, the existence of publication bias could not be conclusively tested. Review Manager software (RevMan v.5.4.1, The Cochrane Collaboration, 2020) was used to summarize the effects and construct the plots. The analysis was performed by three reviews (MJCH, DOV and PMM).

Two reviewers (MJCH and PMM) used the approach proposed by the Grading of Recommendations Assessment, Development and Evaluation (GRADE) [31] to classify the importance of evidence obtained as high, moderate, low or very low and to discern. Factors that could decrease the quality of the evidence were: (1) the study design, (2) the risk of bias, (3) inconsistency in the results, (4) indirect evidence, (5) imprecision and/or (6) other factors. Disagreements were evaluated for a third review (DOV).

## Results

### Studies selection

**Fig. 1:**
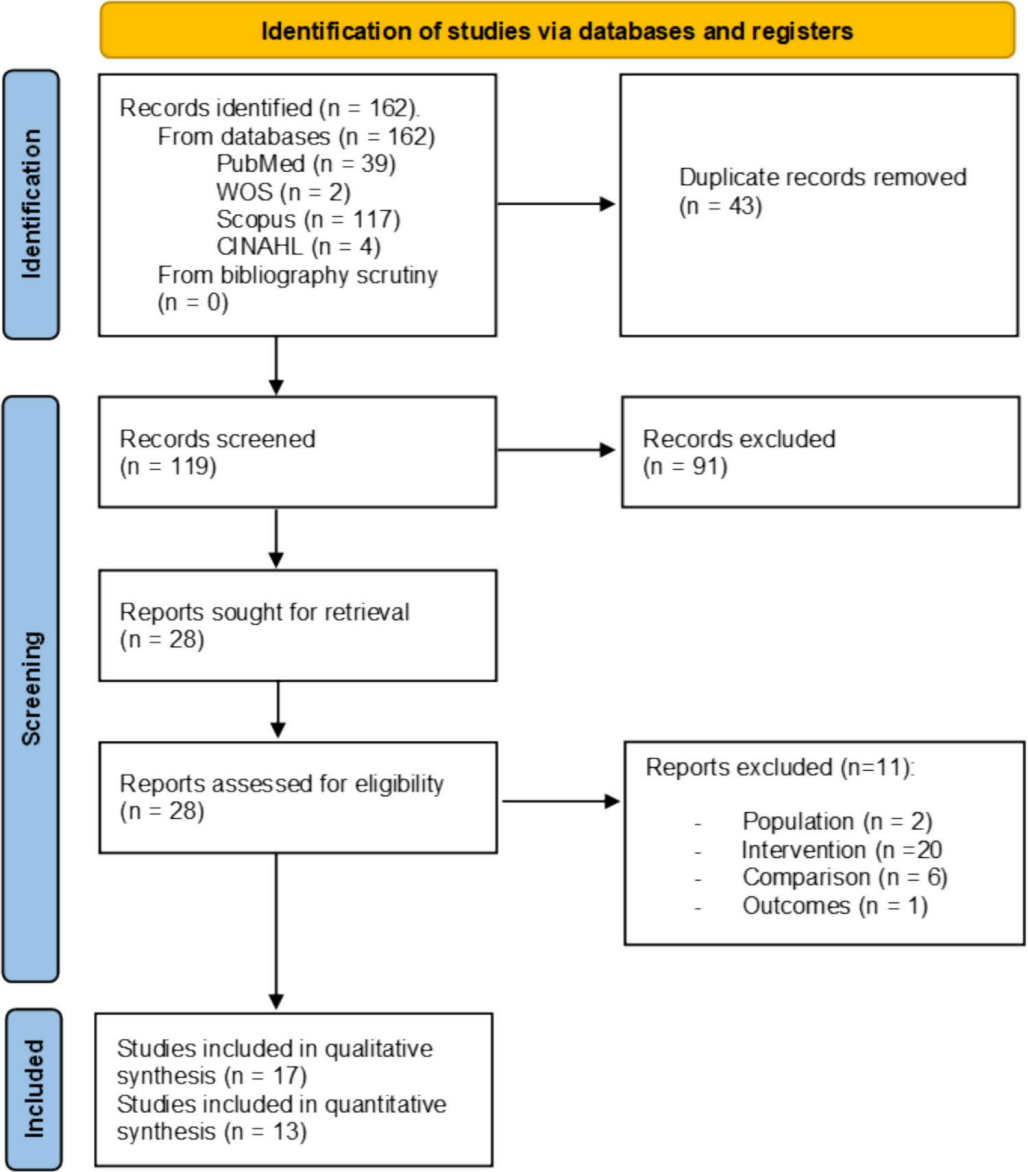
PRISMA flow diagram

After completing the selection process following the PRISMA recommendations, 17 studies were finally included in the qualitative synthesis [21–23, 27, 28, 32–43] and 13 in the quantitative synthesis [21, 22, 32–39, 41–43]. A more detailed description of the selection process is shown in the PRISMA flow diagram (Fig. 1) and in the Supplementary File 2.

### Methodological quality assessment: Risk of Bias tool 2

**Fig. 2:**
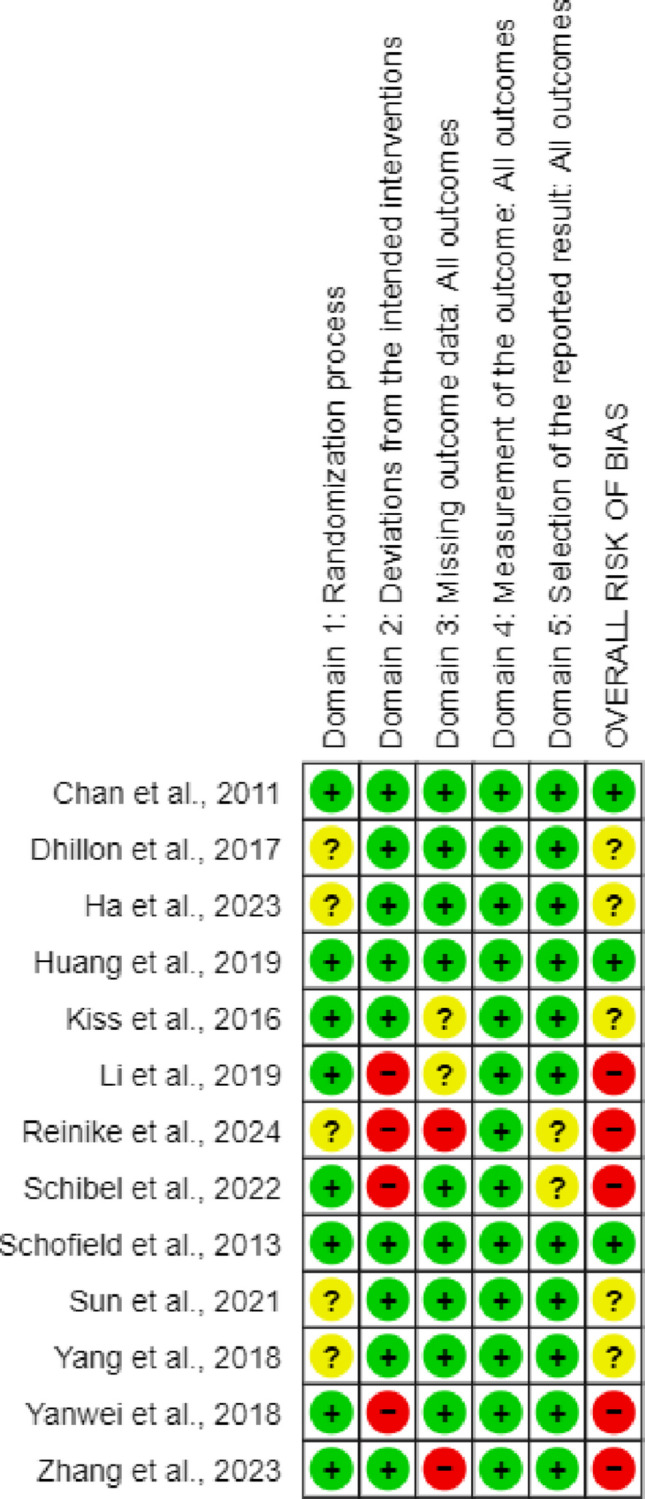
Risk of Bias tool 2

After completing the methodological quality assessment using the Cochrane Risk of Bias tool 2, four studies [22, 35, 37, 42] obtained low risk of bias, five [23, 32, 34, 39, 40] had some concerns, and eight [21, 27, 28, 33, 36, 38, 41, 43], high concerns (Fig. 2). The percentage of discrepancy was approximately 5%.

### The Completeness of Intervention Descriptions

All items were reported by 30% of the clinical trials, and 70% reported at least 3/4 of the items included in the TIDieR checklist. Items 11 (how and by whom was the intervention fidelity assessed) and 12 (description of the extent to which the intervention was delivered as planned) were the least reported, and absent in 47% of the included studies.

### Description of the selected studies

Seventeen RCTs [21–23, 27, 28, 32–43] with a total sample of 1799 adults diagnosed with lung cancer were included. The sample of all studies except Ha et al., 2023 [40] consisted of people diagnosed with lung cancer undergoing treatment. The sample of Ha et al., 2023 [40] consisted of lung cancer survivors. Fifty per cent of the studies had a predominantly female sample, 41.18% had a predominantly male sample and 11.76% had a gender-equal sample. Four modalities of patient education were identified: face-to-face (50% of studies), online (29.42% of studies), telephone (5.88% of studies) and mixed (17.65% of studies). Those that combined different types of resources in the intervention (face-to-face, online and/or telephone) were considered mixed modality. 29.41% of the studies lasted 3 months, 41.18% lasted less than 3 months (2 sessions to 8 weeks), 11.76% lasted more than 3 months (4 months to 12 months) and 11.76% did not specify the duration. The intervention was provided by nurses, multidisciplinary teams, physical exercise specialists or nutritionists. The content of the interventions for all education modalities was homogeny: knowledge about the cancer and its symptoms/sequels and self-management strategies. Occasionally, education was combined with exercise, psychological intervention, homework, or usual care and, in all cases, the control group received usual care or routine intervention. Quality of life was evaluated by 76,47% of the studies, anxiety by 64,71% and fatigue by 29,41%. The results obtained by the studies were controversial: the 53,85%, the 45,45% and the 60% of the studies obtained favourable results for the improvement of quality of life, anxiety and fatigue, respectively. Extended information is shown in Table 1.

**Table 1.**
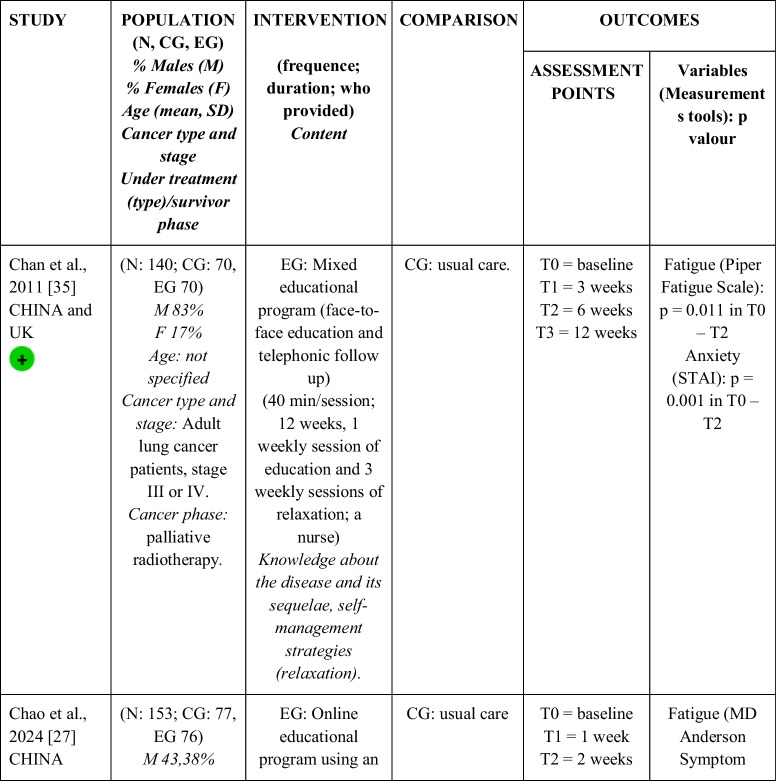

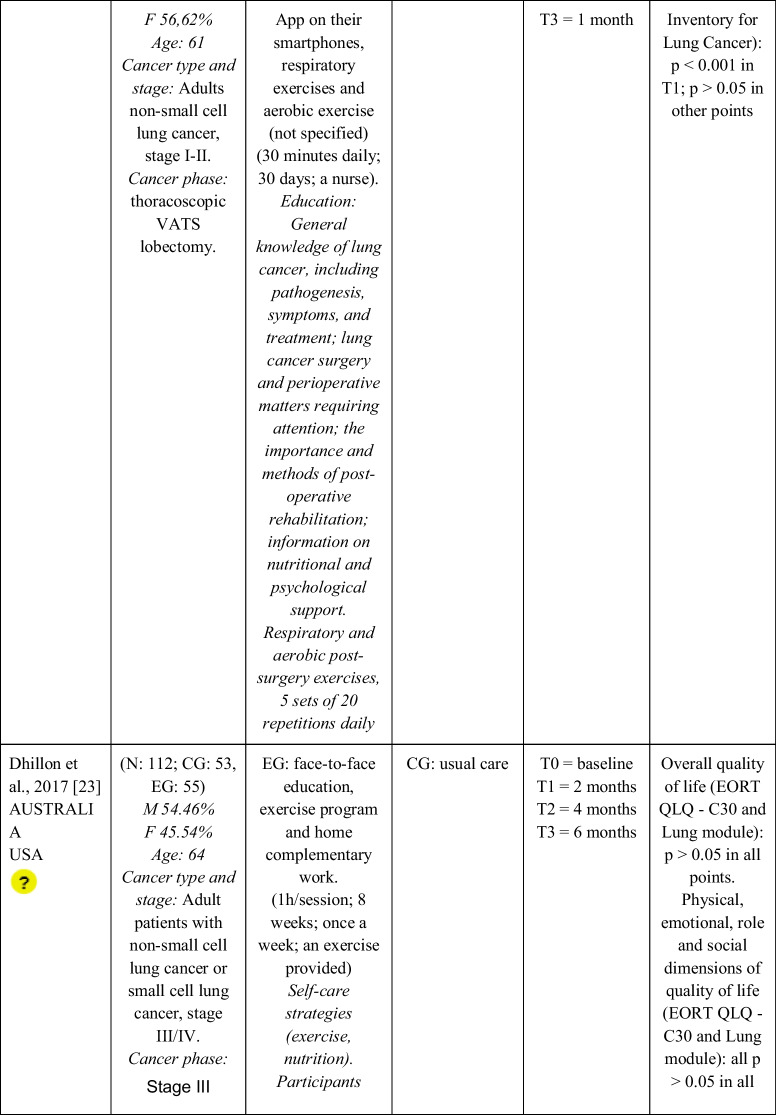

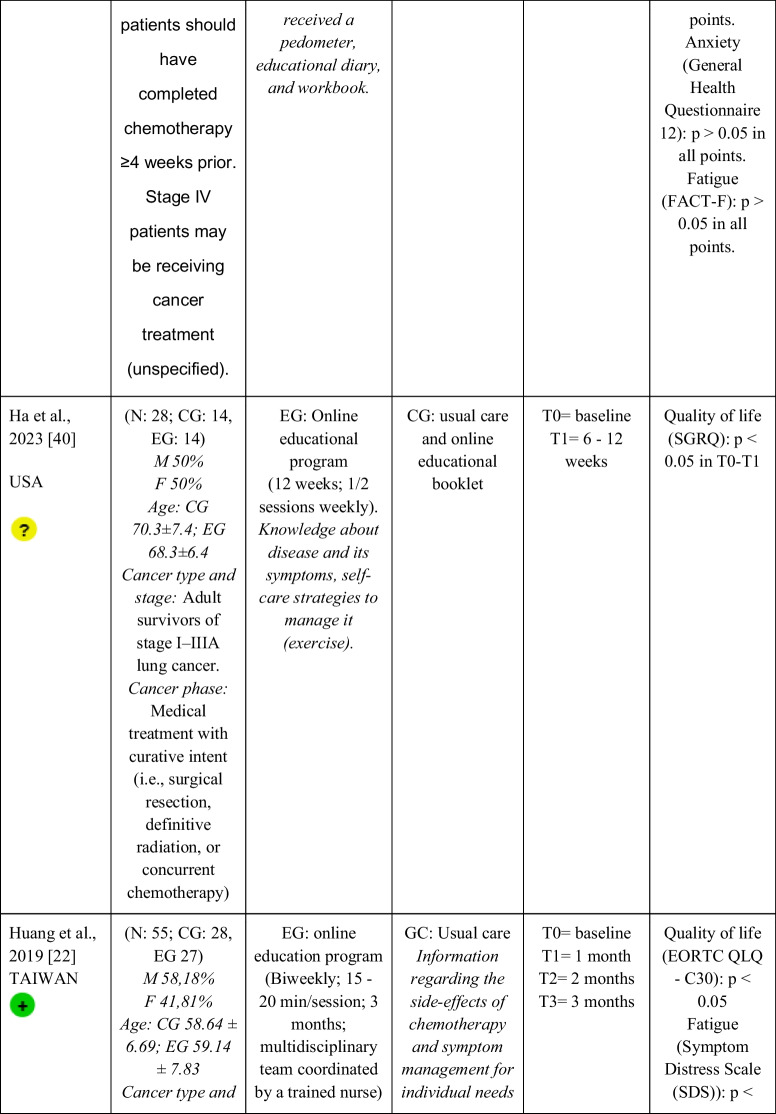

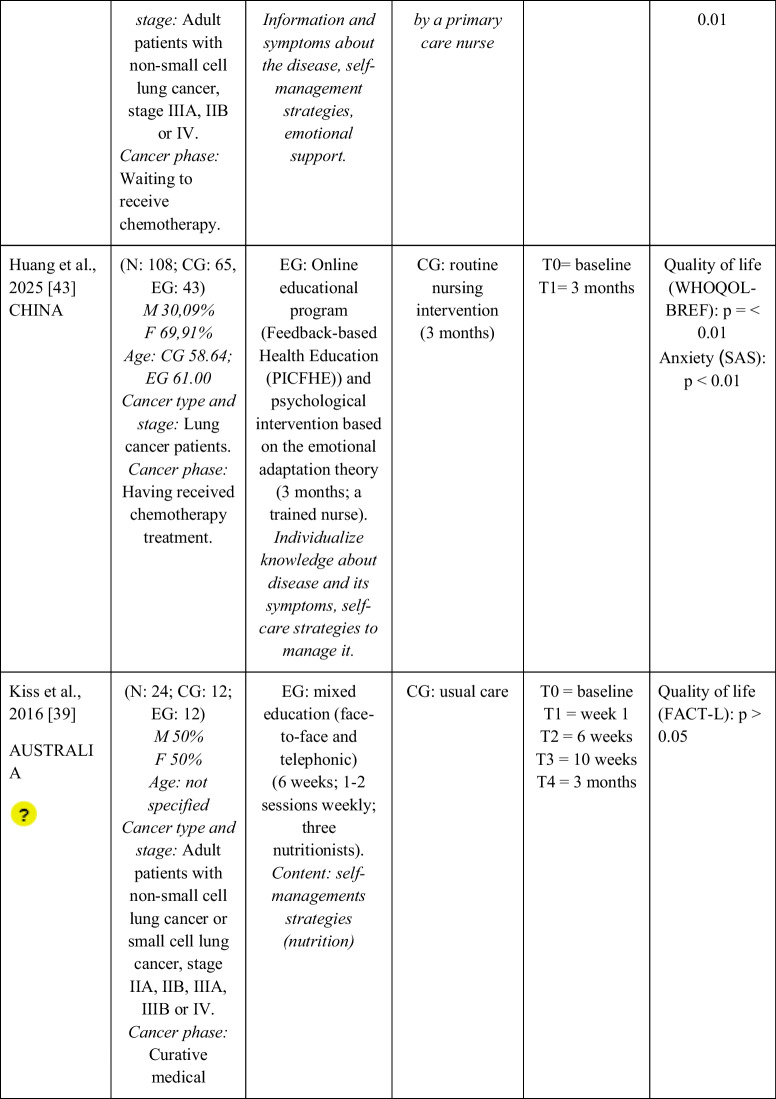

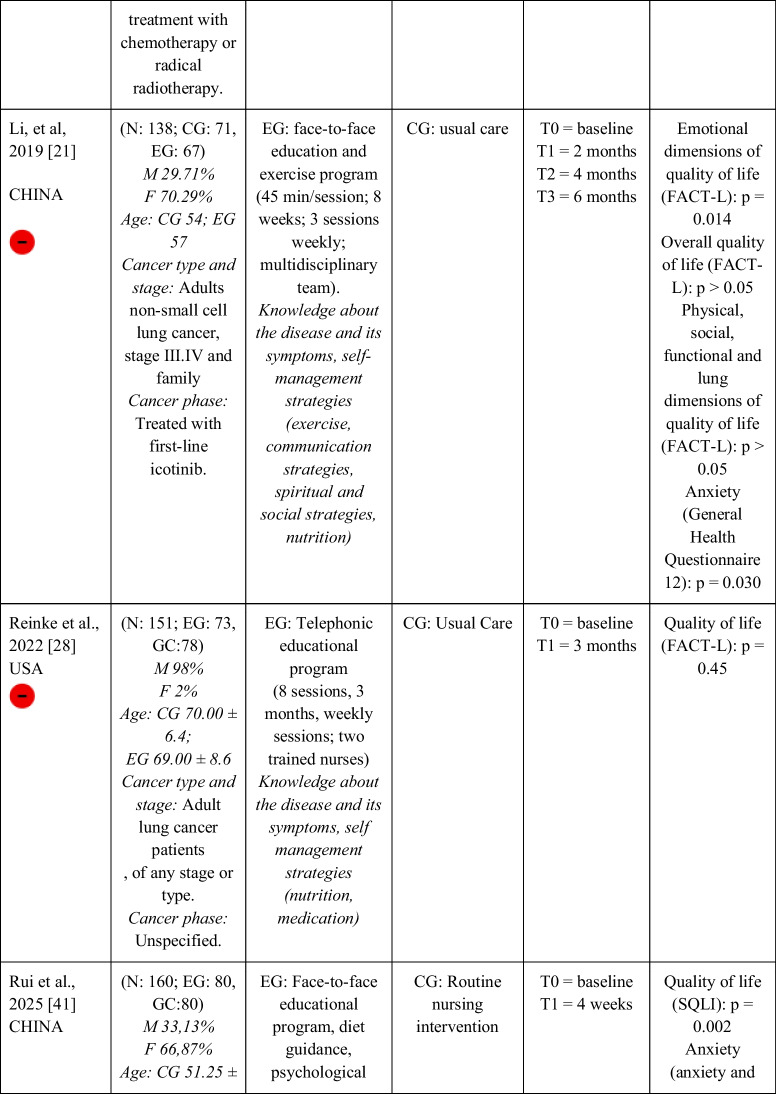

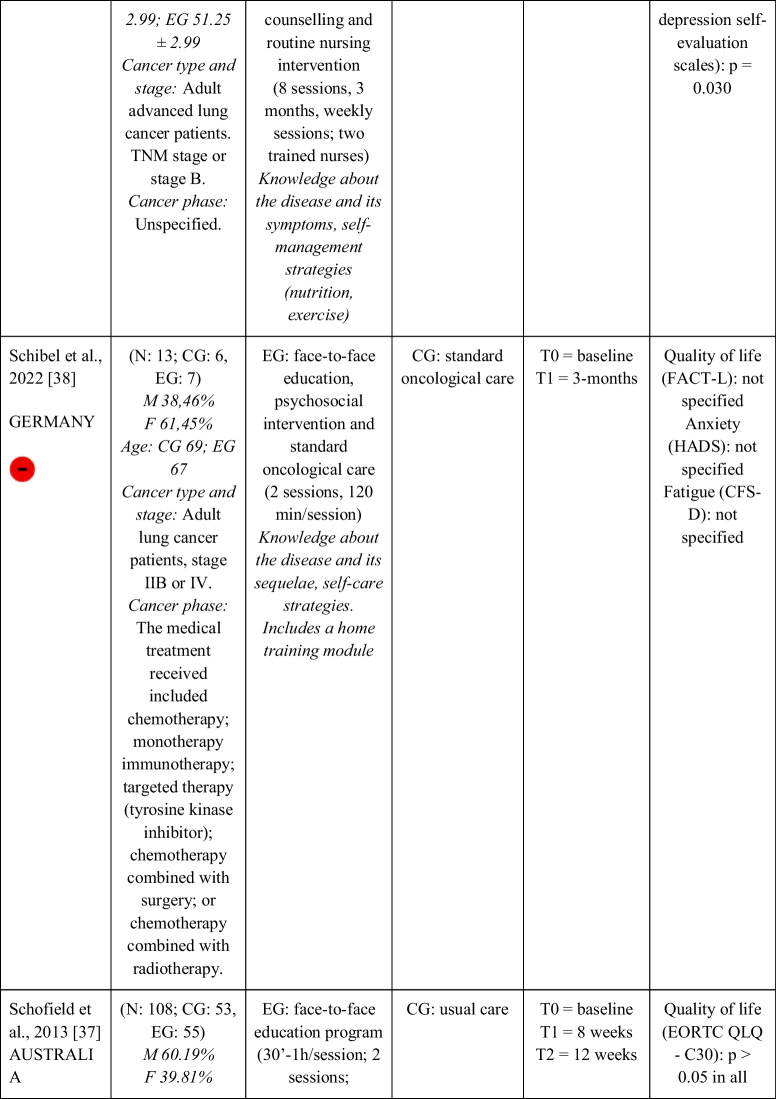

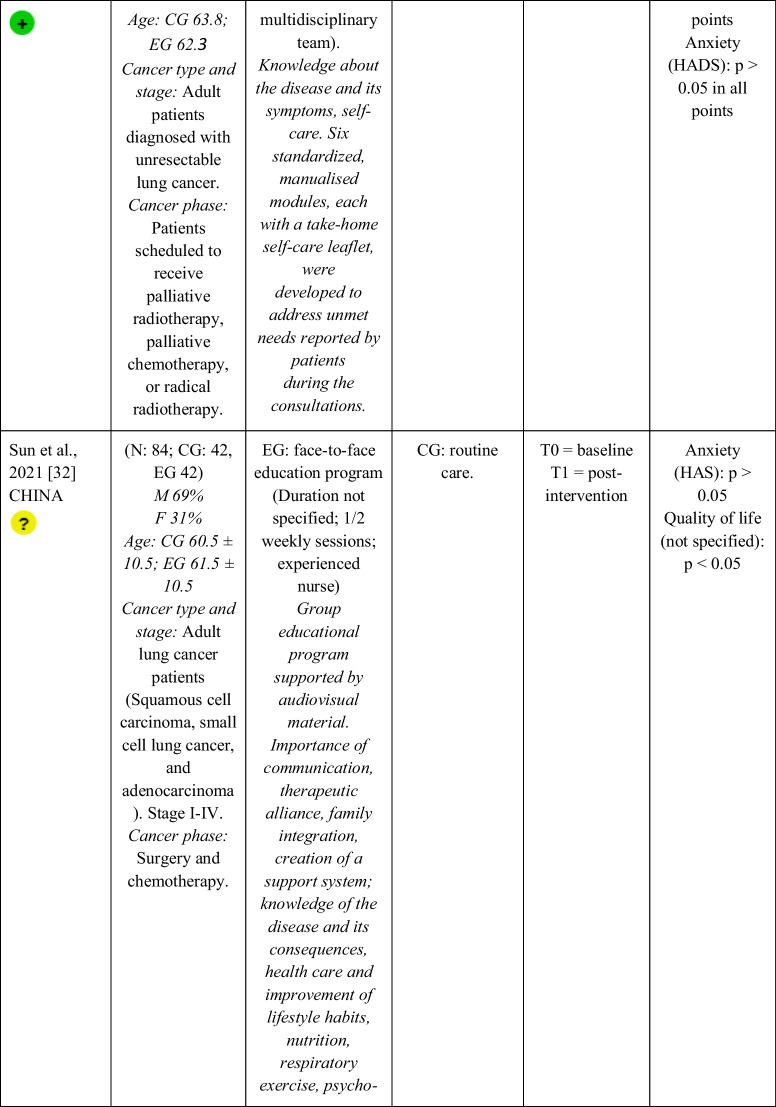

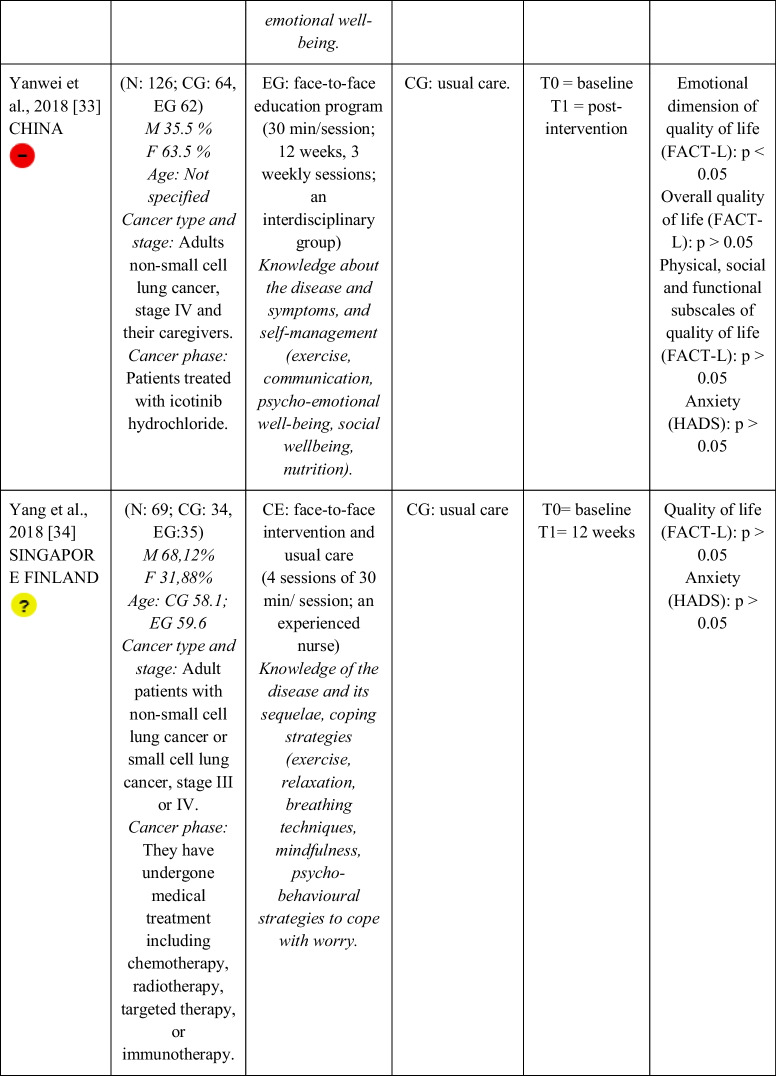

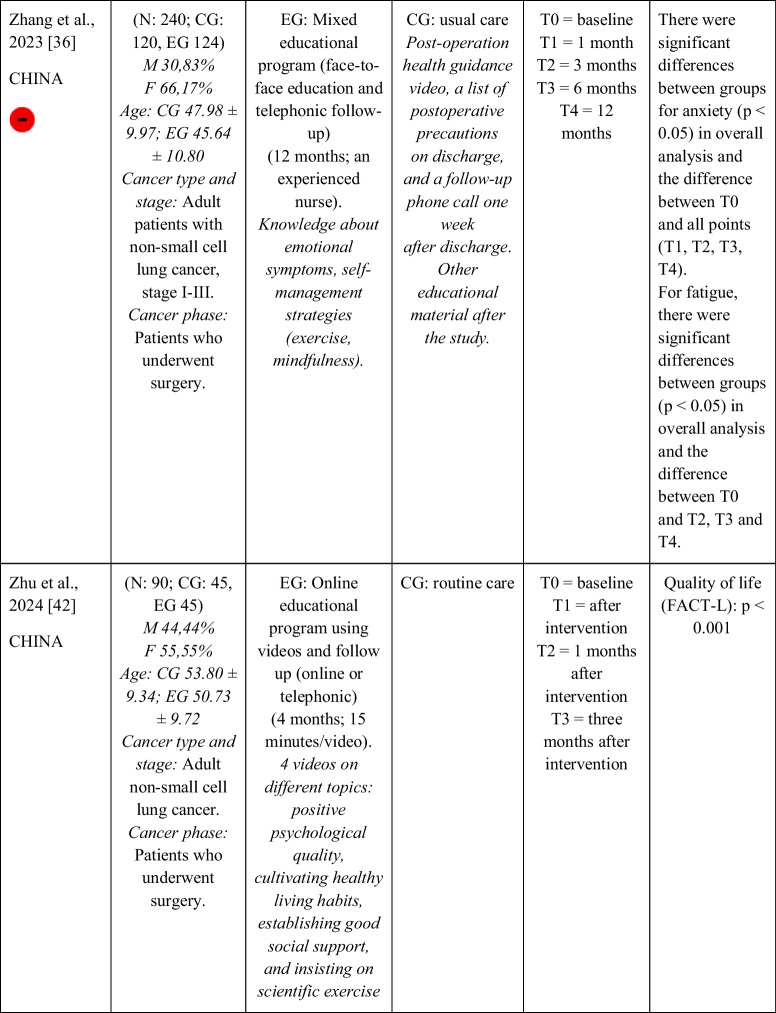
Data extraction

Green: low concerns; yellow: some concerns; red: high concerns. CFS-D: Cancer fatigue scale; CG: control group; EG: experimental group; EORTC QLQ - C30: European Organization for Research and Treatment of Cancer Quality of Life Questionnaire Core 30; F: females; FACIT-F: Functional Assessment of Chronic Illness Therapy-Fatigue Scale; FACT-L: Functional Assessment of Cancer Therapy-Lung; HADS: Hospital Anxiety and Depression Scale; HAS: Hamilton Anxiety Scale; M: males; N: sample; SAS: Self Rating Anxiety Scale; SGRQ: St.-George’s-Respiratory-Questionnaire; SQLI: Spitzer Quality of Life Index ; STAI: A-state scale of the State-Trait Anxiety Inventory; WHOQOL-BREF: World Health Organization Quality of Life Instrument, short form.

### Main outcome: Quality-of-life

**Fig. 3:**
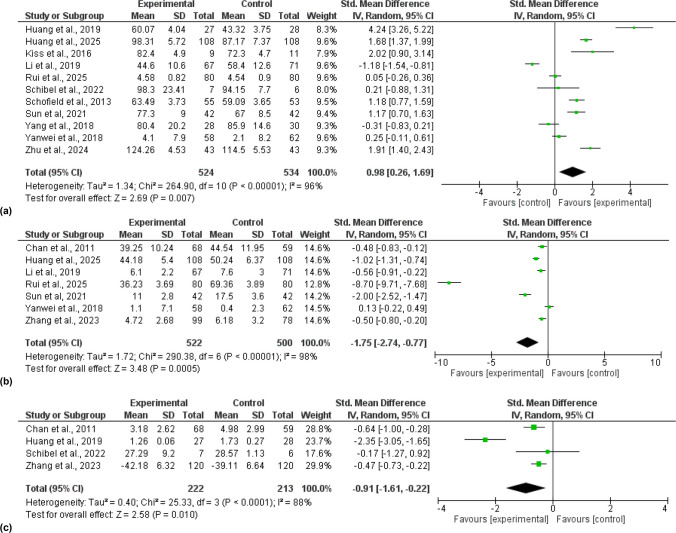
Forests plot: (**a**) quality of life, (**b**) anxiety, (**c**) fatigue

Quality of life was assessed by 14 studies [21–23, 28, 32–34, 37–43], with the most commonly used scales for its assessment being the EORTC-QLQ-C30 and FACT. A meta-analysis was carried out pooling data reported by eleven studies, comparing the application of patient education to the control group. The results obtained are shown in Fig. 3.a. There was a statistically significant difference (Standardized Mean Difference (SMD) = 0.98; 95% CI [0.26, 1.69], p = 0.007, I^2^ = 96%) and a large effect size for this outcome in favour of the experimental groups. The sensitivity analysis showed how heterogeneity decreased (I^2^ = 93%) after excluding Li et al. (2019) [21] and Huang et al. (2019) [22], but heterogeneity remained high. Supplementary File 3 (Figure SF3.a.) shows the funnel plot for this meta-analysis.

### Secondary outcomes: Anxiety and Fatigue

Anxiety was assessed by 11 studies [21, 23, 32–38, 41, 43], and fatigue by 6 [22, 23, 27, 35, 36, 38]. Two meta-analyses were conducted, one for each outcome, pooling data reported by seven and four trials, respectively. The results obtained are shown in Fig. 3.b. and Fig. 3.c As it can be observed, there were statistically significant differences and a large effect size in favour of the experimental intervention for anxiety and fatigue (Standardized Mean Difference (SMD) = −1.75; 95% CI [−2.74, −0.77], p = 0.0005, I2 = 98%; Standardized Mean Difference (SMD) = −0.091; 95% CI [−1.61, −0.22], p = 0.01, I2 = 88%, respectively). The sensitivity analysis showed how heterogeneity decreased for anxiety (I^2^ = 84%) after excluding Rui et al. (2025) [41] and Sun et al. (2021) [32], although heterogeneity remained high. In contrast, heterogeneity was not observed for fatigue when Huang et al. (2019) [22] was excluded in the sensitivity analysis. Supplementary File 3 (Figure SF3.b.; Figure SF3.c.) shows the funnel plots for each meta-analysis.

### Evidence synthesis

Evidence synthesis was carried out using the GRADE system and considering quality of life, anxiety and fatigue outcomes (Table 2). The results were classified with a very low level of evidence and were associated with a critical level of importance.

**Table 2:** Certainty of evidence (GRADE)

Summary of findings			Certainty in evidence based on the GRADE approach						
Outcome	Number and type of studies	Participants (EG, CG)	Risk of bias	Inconsistency	Indirectness	Imprecision	Publication bias	Level of evidence	Importance
Quality of life	11 (RCTs)	1,058 (524, 534)	Serious (−1)^a^	Very serious (−2)^b^	Not serious	Not serious	Undetected	Very low ⨁◯◯◯	Critical
Anxiety	7 (RCTs)	1,022 (522, 500)	Very serious (−2)^c^	Very serious (−2)^b^	Not serious	Not serious	Undetected	Very low ⨁◯◯◯	Critical
Fatigue	4 (RCTs)	425 (222, 213)	Very serious (−2)^c^	Serious (−1)^d^	Not serious	Not serious	Undetected	Very low ⨁◯◯◯	Critical

Note: GRADE = Grading of Recommendations Assessment, Development and Evaluation. CG: control group; EG: experimental group; N: sample of primary studies in meta-analysis not considering the multi-arm; n: number of studies; k: number of arms of studies included in meta-analysis; S: complete sample included in the meta-analysis considering all multi-arms. The results of GRADE were based on the meta-analyses findings

a. Risk of bias: one level was downgraded if at least 25% of the included studies reported high risk of bias.

b. Inconsistency: two levels were downgraded if high heterogeneity was detected after sensitivity analysis (I2 > 50%).

c. Risk of bias: two levels were downgraded if at least 50% of the included studies reported high risk of bias.

d. Inconsistency: one level was downgraded if high heterogeneity was detected before sensitivity analysis (I2 > 50%).

## Discussion

This review and meta-analysis aimed to synthesise the evidence regarding the effect of patient education on quality of life, fatigue, and pain in individuals diagnosed with lung cancer. Across all included studies, statistically significant differences and large effect sizes were identified in favour of patient education interventions. These findings suggest that educational strategies may serve as effective adjuncts in the management of symptoms commonly experienced by this population. However, a high degree of heterogeneity was observed in all analyses. While this variation limits the generalisability of the findings, the consistent direction of effect across studies lends support to the robustness of the overall conclusions. These results align with previous studies on lung cancer [44–49], which indicate that patient education can lead to improvements in quality of life, anxiety, and fatigue. Furthermore, our findings are in line with systematic reviews in other cancer populations, such as breast and colorectal cancer [11–17], which highlight education as an effective tool for managing cancer-related symptoms and sequelae.

As underscored by the World Health Organization [50] and Ellis’s ABC model [51], individuals vary in their coping strategies when facing health-related stressors. Nevertheless, coping mechanisms play a critical role in shaping the impact of illness on quality of life[50, 51]. Patient education functions as a therapeutic approach aimed at enhancing self-management and adaptive coping, adopting a holistic perspective that integrates emotional, behavioural, and cognitive components [7–11]. According to the World Health Organization, it is a key strategy for empowerment and quality of life improvement in chronic conditions [7–11, 50]. Since lung cancer is associated with persistent symptoms such as fatigue and anxiety, which can compromise quality of life for prolonged periods [4], the observed benefits of education likely reflect its potential to enhance health literacy, foster comprehensive coping, and facilitate behaviour change.

On the other hand, the substantial heterogeneity observed may stem from two main categories of factors. The first relates to prognostic variability — that is, differences in clinical characteristics that influence the course of the disease, such as cancer type, stage, treatment modality, and the phase of the illness. The second concerns variability in the educational interventions themselves, including differences in format, duration, timing in relation to the cancer trajectory, integration with other therapeutic approaches, methodological design, educational framework, and the professional background of the educator.

As previously mentioned, one of the main limitations of this review is the considerable heterogeneity observed across studies. While this was formally addressed when assessing the certainty of evidence using the GRADE framework, the limited number of available trials precluded the possibility of conducting subgroup analyses that might have helped to explain and reduce such variability. This, in turn, restricts the interpretability and generalisability of the findings. Moreover, the moderate risk of bias identified in several studies further weakens the strength of the conclusions. Variability in the quality and completeness of reporting also limited the extraction of key methodological details, potentially affecting the robustness of the synthesis. Finally, the search strategy employed may have inadvertently excluded potentially relevant studies, thereby introducing a risk of selection bias.

Nevertheless, this review is the first to systematically consolidate current evidence regarding the efficacy of therapeutic education in lung cancer, highlighting its clinical potential. Our findings provide valuable insights for both clinical practice and future research. In the clinical context, they can inform the development of more up-to-date and evidence-based educational programmes aimed at improving patient care. From a research perspective, this review reinforces the need for high-quality, low-bias clinical trials that allow for subgroup analyses to reduce heterogeneity and explore specific intervention components. Moreover, grounding future interventions in established theoretical frameworks and incorporating standardised patient-reported outcomes could enhance their rigour and relevance. Ensuring greater demographic diversity in future samples would also contribute to broader applicability. Furthermore, this review serves as a foundation for future systematic reviews exploring educational interventions in other cancer types, or assessing their impact on a wider range of symptoms and functional outcomes in lung cancer.

## Conclusion

The evidence to date regarding the effects of patient education interventions on individuals diagnosed with lung cancer seems to suggest that they improve quality of life, reduce anxiety and alleviate fatigue. However, these findings should be interpreted with caution due to the limited number of studies, their moderate risk of bias, and their heterogeneity, which collectively result in a level of evidence classified as ‘very low’. It is recommended that further studies be conducted on this subject to clarify the effectiveness of patient education within this population.

## Supplementary Information

Below is the link to the electronic supplementary material.

Supplementary file1 (DOCX 2587 KB)

Supplementary file2 (DOCX 56 KB)

## Data Availability

The datasets analyzed during the current study are not publicly available. Specific data or further details can be requested from the corresponding author.
