# Supplementary material for: Effect of patient education in improving quality of life, fatigue and anxiety in people diagnosed with lung cancer: systematic review
Source: Support Care Cancer. 2026 Feb 3;34(2):159. doi: 10.1007/s00520-026-10331-8 (PMC12868036; doi:10.1007/s00520-026-10331-8)

**Supplementary File 1** - Detailed search Strategy in PubMed

Search: **("lung neoplasms"[MeSH Terms] AND ("health education"[MeSH Terms] OR "educat*"[Title/Abstract]) AND ("quality of life"[MeSH Terms] OR "fatigue"[MeSH Terms] OR "anxiety"[MeSH Terms])**

Filters: **Clinical Trial, Randomized Controlled Trial**

("lung neoplasms"[MeSH Terms] AND ("health education"[MeSH Terms] OR "educat*"[Title/Abstract]) AND ("quality of life"[MeSH Terms] OR "fatigue"[MeSH Terms] OR "anxiety"[MeSH Terms])) AND (clinicaltrial[Filter] OR randomizedcontrolledtrial[Filter])

**Supplementary File 2** - Detailed selection process

The study selection process followed the PRISMA guidelines and flow diagram model. A total of 162 records were identified through database searching: PubMed (n = 39), Web of Science (n = 2), Scopus (n = 117), and CINAHL (n = 4). No additional records were identified through reference list screening. After removing duplicates (n = 43), 119 records remained for screening. These were assessed based on title and abstract, resulting in the exclusion of 91 records. The full texts of the remaining 28 studies were assessed for eligibility. Ultimately, 11 studies were excluded, leaving 17 included in the qualitative synthesis and 13 in the quantitative analysis. Table SF2.1 provides a detailed account of the reasons for exclusion of the 11 studies.

**Table SF2.1**. Detailed studies’ reason of exclusion

| **Studies** | **Exclusion by** | **Reasons** |
| --- | --- | --- |
| Howell D. et al, 2023 | **Population** | The sample were formed by patients with different types of cancer diagnosis (not only lung cancer). |
| Miura S. et al, 2019 |  |  |
| Takano T. et al 2021 | **Intervention** | The intervention did not fit the definition of education considered by this study and explained in the introduction. |
| Yorke J. et al, 2015 |  |  |
| Fugazzaro S. et al, 2017 | **Comparison** | Control also received education. |
| Mosher C. et al, 2016 |  |  |
| Mosher C. et al, 2019 |  |  |
| Tenconi S. et al, 2021 |  |  |
| Wagnum K. et al, 2013 |  |  |
| Zhu J. et al, 2024 |  |  |
| Wilkie D. et al, 2010 | **Outcomes** | The study don’t consider none of the established variables. |

Fugazzaro, S., Costi, S., Mainini, C., Kopliku, B., Rapicetta, C., Piro, R., Bardelli, R., Rebelo, P. F. S., Galeone, C., Sgarbi, G., Lococo, F., Paci, M., Ricchetti, T., Cavuto, S., Merlo, D. F., & Tenconi, S. (2017). PUREAIR protocol: Randomized controlled trial of intensive pulmonary rehabilitation versus standard care in patients undergoing surgical resection for lung cancer. *BMC Cancer, 17*(1). Scopus.<https://doi.org/10.1186/s12885-017-3479-y>

Miura, S., Naito, T., Mitsunaga, S., Omae, K., Mori, K., Inano, T., Yamaguchi, T., Tatematsu, N., Okayama, T., Morikawa, A., Mouri, T., Tanaka, H., Kimura, M., Imai, H., Mizukami, T., Imoto, A., Kondoh, C., Shiotsu, S., Okuyama, H., … Takayama, K. (2019). A randomized phase II study of nutritional and exercise treatment for elderly patients with advanced non-small cell lung or pancreatic cancer: The NEXTAC-TWO study protocol. *BMC Cancer, 19*(1). Scopus.<https://doi.org/10.1186/s12885-019-5762-6>

Howell, D., Pond, G. R., Bryant-Lukosius, D., Powis, M., McGowan, P. T., Makuwaza, T., Kukreti, V., Rask, S., Hack, S., & Krzyzanowska, M. K. (2023). Feasibility and effectiveness of self-management education and coaching on patient activation for managing cancer treatment toxicities. *Journal of the National Comprehensive Cancer Network: JNCCN, 21*(3), 247-256.e8.<https://doi.org/10.6004/jnccn.2022.7095>

Mosher, C. E., Secinti, E., Hirsh, A. T., Hanna, N., Enhorn, L. H., Jalal, S. I., Durm, G., Champion, V. L., & Johns, S. A. (2019). Acceptance and commitment therapy for symptom interference in advanced lung cancer and caregiver distress: A pilot randomized trial. *Journal of Pain and Symptom Management, 58*(4), 632-644.<https://doi.org/10.1016/j.jpainsymman.2019.06.021>

Mosher, C. E., Winger, J. G., Hanna, N., Jalal, S. I., Einhorn, L. H., Birdas, T. J., Ceppa, D. P., Kesler, K. A., Schmitt, J., Kashy, D. A., & Champion, V. L. (2016). Randomized pilot trial of a telephone symptom management intervention for symptomatic lung cancer patients and their family caregivers. *Journal of Pain and Symptom Management, 52*(4), 469-482. Scopus.<https://doi.org/10.1016/j.jpainsymman.2016.04.006>

Takano, T., Matsuda, A., Ishizuka, N., Ozaki, Y., Suyama, K., Tanabe, Y., Miura, Y., & Matsushima, E. (2021). Effectiveness of self-help workbook intervention on quality of life in cancer patients receiving chemotherapy: Results of a randomized controlled trial. *BMC Cancer, 21*(1), 1-9.

Tenconi, S., Mainini, C., Rapicetta, C., Braglia, L., Galeone, C., Cavuto, S., Merlo, D. F., Costi, S., Paci, M., Piro, R., & Fugazzaro, S. (2021). Rehabilitation for lung cancer patients undergoing surgery: Results of the PUREAIR randomized trial. *European Journal of Physical and Rehabilitation Medicine, 57*(6), 1002-1011. Scopus.<https://doi.org/10.23736/S1973-9087.21.06789-7>

Wangnum, K., Thanarojanawanich, T., Chinwatanachai, K., Jamprasert, L., Maleehuan, O., & Janthakun, V. (2013). Impact of the multidisciplinary education program in self-care on fatigue in lung cancer patients receiving chemotherapy. *Journal of the Medical Association of Thailand, 96*(12), 1601-1608.

Wilkie, D., Berry, D., Cain, K., Huang, H.-Y., Mekwa, J., Lewis, F., Gallucci, B., Lin, Y.-C., Chen, A. C.-C., & Ko, N.-Y. (2010). Effects of coaching patients with lung cancer to report cancer pain. *Western Journal of Nursing Research, 32*(1), 23-46. Scopus.<https://doi.org/10.1177/0193945909348009>

Yorke, J., Lloyd-Williams, M., Smith, J., Blackhall, F., Harle, A., Warden, J., Ellis, J., Pilling, M., Haines, J., Luker, K., & Molassiotis, A. (2015). Management of the respiratory distress symptom cluster in lung cancer: A randomised controlled feasibility trial. *Support Care Cancer, 23*(11), 3373-3384. https://doi.org/10.1007/s00520-015-2810-x

Zhu, J., Chen, S.-H., Guo, J.-Y., Li, W., Li, X.-T., Huang, L.-H., & Ye, M. (2024). Effect of digital storytelling intervention on resilience, self-efficacy and quality of life among patients with non-small cell lung cancer (NSCLC): A randomized controlled trial. *European Journal of Oncology Nursing: The Official Journal of European Oncology Nursing Society, 69*, 102535.<https://doi.org/10.1016/j.ejon.2024.102535>

**Supplementary File 3** - Funnel plots

**Figure SF3. a.** Funnel plot: quality of life


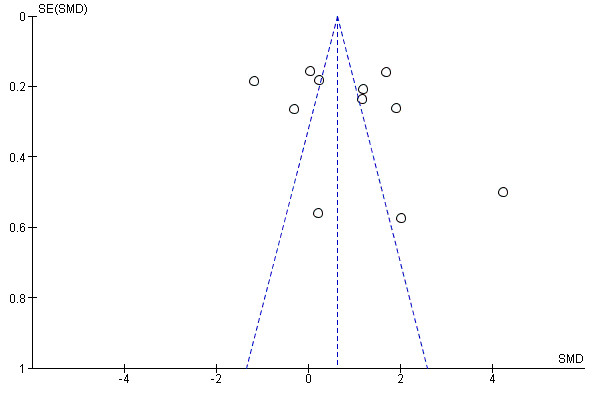


**Figure SF3. b.** Funnel plot: anxiety


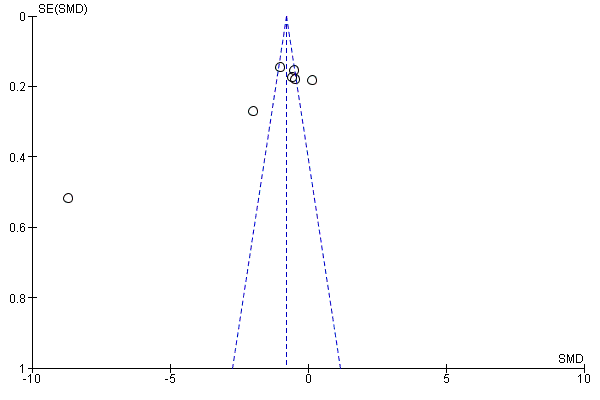


**Figure SF3. c.** Funnel plot: fatigue


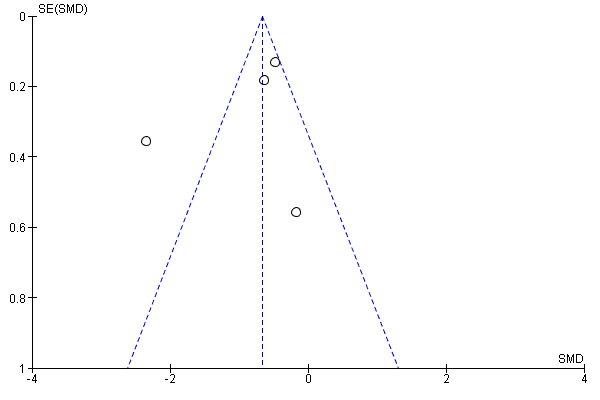

Supplement: Supplementary file 1 — Supplementary file1 (DOCX 2587 KB) [file 520_2026_10331_MOESM1_ESM.docx]
