# Supplementary material for: Effect of patient education in improving quality of life, fatigue and anxiety in people diagnosed with lung cancer: systematic review
Source: Support Care Cancer. 2026 Feb 3;34(2):159. doi: 10.1007/s00520-026-10331-8 (PMC12868036; doi:10.1007/s00520-026-10331-8)

**SUPPLEMENTARY MATERIAL A** - Detailed search Strategy

PUBMED (217 potential articles): **'lung neoplasms'[MeSH Terms] AND ('health education'[MeSH Terms] OR 'educat*'[Title/Abstract]) AND ('quality of life'[MeSH Terms] OR 'fatigue' [MeSH Terms] OR 'anxiety' [MeSH Terms])**

"lung neoplasms"[MeSH Terms] AND ("health education"[MeSH Terms] OR "educat*"[Title/Abstract]) AND ("quality of life"[MeSH Terms] OR "fatigue"[MeSH Terms] OR "anxiety"[MeSH Terms])

**Translations**

'lung neoplasms'[MeSH Terms]: "lung neoplasms"[MeSH Terms]

'health education'[MeSH Terms]: "health education"[MeSH Terms]

'quality of life'[MeSH Terms]: "quality of life"[MeSH Terms]

'fatigue' [MeSH Terms]: "fatigue"[MeSH Terms]

'anxiety' [MeSH Terms]: "anxiety"[MeSH Terms]

**SUPPLEMENTARY MATERIAL B** - Detailed selection process

The studies selection process followed the PRISMA recommendations and flow diagram model. In the data sources, 217 results were found. Duplicates were removed, obtaining 46 results. These results were reviewed only by title reading and those who did not meet the subject of this review were excluded. After this, 36 were analysed by reading titles and abstracts, excluding 12 articles and obtaining 24 to analyse in the next step. After reviewing the 24 full texts, 11 studies were removed, including 13 articles in the qualitative synthesis and 11 in the quantitative synthesis. Eleven studies were excluded after reviewing the full text for various reasons related with the inclusion and exclusion criteria:

- Two studies were excluded related to the population established in the PICOS question. The reason for the exclusion was that the groups were formed by patients with different types of cancer diagnosis, and not only lung cancer (IC.P reason).
- Two studies were excluded related to the intervention established in the PICOS question. This was because the intervention that was carried out did not fit the definition of education considered by this study and explained in the introduction (IC.I reason).
- Six studies were excluded related to the comparison established in the PICOS question, since both groups (control and intervention group) received education (IC.C reason).
- One study was excluded related to the outcome established in the PICOS question, given that the established variables for this review were not measured in it (IC.O reason).

The studies excluded during the full-text reviewing phase are showed in table B1, where the exclusion reasons are summarised.

**Table B1**. Studies excluded for full-text

| **Studies** | **Exclusion reasons** |
| --- | --- |
| Howell D. et al, 2023 [3] | **IC. P** |
| Miura S. et al, 2019 [2] |  |
| Takano T. et al 2021 [6] | **IC. I** |
| Yorke J. et al, 2015 [10] |  |
| Fugazzaro S. et al, 2017 [1] | **IC.C** |
| Mosher C. et al, 2016 [5] |  |
| Mosher C. et al, 2019 [4] |  |
| Tenconi S. et al, 2021 [7] |  |
| Wagnum K. et al, 2013 [8] |  |
| Zhu J. et al, 2024 [11] |  |
| Wilkie D. et al, 2010 [9] | **IC. O** |

IC. P: studies excluded by the population reason; IC.I: studies excluded by the intervention reason; IC.C: studies excluded by the comparison reason; IC.O studies excluded by the outcome reason.

**Complete references**

1. Fugazzaro, S., Costi, S., Mainini, C., Kopliku, B., Rapicetta, C., Piro, R., Bardelli, R., Rebelo, P. F. S., Galeone, C., Sgarbi, G., Lococo, F., Paci, M., Ricchetti, T., Cavuto, S., Merlo, D. F., & Tenconi, S. (2017). PUREAIR protocol: Randomized controlled trial of intensive pulmonary rehabilitation versus standard care in patients undergoing surgical resection for lung cancer. *BMC Cancer, 17*(1). Scopus.<https://doi.org/10.1186/s12885-017-3479-y>
2. Miura, S., Naito, T., Mitsunaga, S., Omae, K., Mori, K., Inano, T., Yamaguchi, T., Tatematsu, N., Okayama, T., Morikawa, A., Mouri, T., Tanaka, H., Kimura, M., Imai, H., Mizukami, T., Imoto, A., Kondoh, C., Shiotsu, S., Okuyama, H., … Takayama, K. (2019). A randomized phase II study of nutritional and exercise treatment for elderly patients with advanced non-small cell lung or pancreatic cancer: The NEXTAC-TWO study protocol. *BMC Cancer, 19*(1). Scopus.<https://doi.org/10.1186/s12885-019-5762-6>
3. Howell, D., Pond, G. R., Bryant-Lukosius, D., Powis, M., McGowan, P. T., Makuwaza, T., Kukreti, V., Rask, S., Hack, S., & Krzyzanowska, M. K. (2023). Feasibility and effectiveness of self-management education and coaching on patient activation for managing cancer treatment toxicities. *Journal of the National Comprehensive Cancer Network: JNCCN, 21*(3), 247-256.e8.<https://doi.org/10.6004/jnccn.2022.7095>
4. Mosher, C. E., Secinti, E., Hirsh, A. T., Hanna, N., Enhorn, L. H., Jalal, S. I., Durm, G., Champion, V. L., & Johns, S. A. (2019). Acceptance and commitment therapy for symptom interference in advanced lung cancer and caregiver distress: A pilot randomized trial. *Journal of Pain and Symptom Management, 58*(4), 632-644.<https://doi.org/10.1016/j.jpainsymman.2019.06.021>
5. Mosher, C. E., Winger, J. G., Hanna, N., Jalal, S. I., Einhorn, L. H., Birdas, T. J., Ceppa, D. P., Kesler, K. A., Schmitt, J., Kashy, D. A., & Champion, V. L. (2016). Randomized pilot trial of a telephone symptom management intervention for symptomatic lung cancer patients and their family caregivers. *Journal of Pain and Symptom Management, 52*(4), 469-482. Scopus.<https://doi.org/10.1016/j.jpainsymman.2016.04.006>
6. Takano, T., Matsuda, A., Ishizuka, N., Ozaki, Y., Suyama, K., Tanabe, Y., Miura, Y., & Matsushima, E. (2021). Effectiveness of self-help workbook intervention on quality of life in cancer patients receiving chemotherapy: Results of a randomized controlled trial. *BMC Cancer, 21*(1), 1-9.
7. Tenconi, S., Mainini, C., Rapicetta, C., Braglia, L., Galeone, C., Cavuto, S., Merlo, D. F., Costi, S., Paci, M., Piro, R., & Fugazzaro, S. (2021). Rehabilitation for lung cancer patients undergoing surgery: Results of the PUREAIR randomized trial. *European Journal of Physical and Rehabilitation Medicine, 57*(6), 1002-1011. Scopus.<https://doi.org/10.23736/S1973-9087.21.06789-7>
8. Wangnum, K., Thanarojanawanich, T., Chinwatanachai, K., Jamprasert, L., Maleehuan, O., & Janthakun, V. (2013). Impact of the multidisciplinary education program in self-care on fatigue in lung cancer patients receiving chemotherapy. *Journal of the Medical Association of Thailand, 96*(12), 1601-1608.
9. Wilkie, D., Berry, D., Cain, K., Huang, H.-Y., Mekwa, J., Lewis, F., Gallucci, B., Lin, Y.-C., Chen, A. C.-C., & Ko, N.-Y. (2010). Effects of coaching patients with lung cancer to report cancer pain. *Western Journal of Nursing Research, 32*(1), 23-46. Scopus.<https://doi.org/10.1177/0193945909348009>
10. Yorke, J., Lloyd-Williams, M., Smith, J., Blackhall, F., Harle, A., Warden, J., Ellis, J., Pilling, M., Haines, J., Luker, K., & Molassiotis, A. (2015). Management of the respiratory distress symptom cluster in lung cancer: A randomised controlled feasibility trial. *Support Care Cancer, 23*(11), 3373-3384. https://doi.org/10.1007/s00520-015-2810-x
11. Zhu, J., Chen, S.-H., Guo, J.-Y., Li, W., Li, X.-T., Huang, L.-H., & Ye, M. (2024). Effect of digital storytelling intervention on resilience, self-efficacy and quality of life among patients with non-small cell lung cancer (NSCLC): A randomized controlled trial. *European Journal of Oncology Nursing: The Official Journal of European Oncology Nursing Society, 69*, 102535.<https://doi.org/10.1016/j.ejon.2024.102535>

**SUPPLEMENTARY MATERIAL C** - Funnel plots

**Figure S.m. C.a.** Funnel plot: quality of life


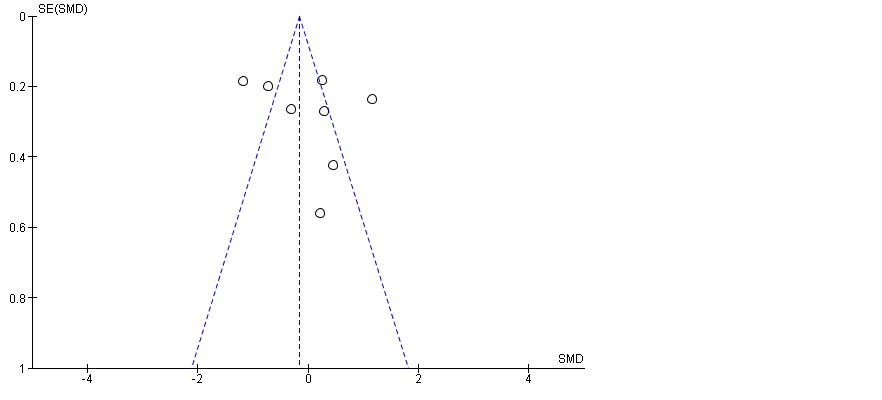


**Figure S.m. C.b.** Funnel plot: anxiety


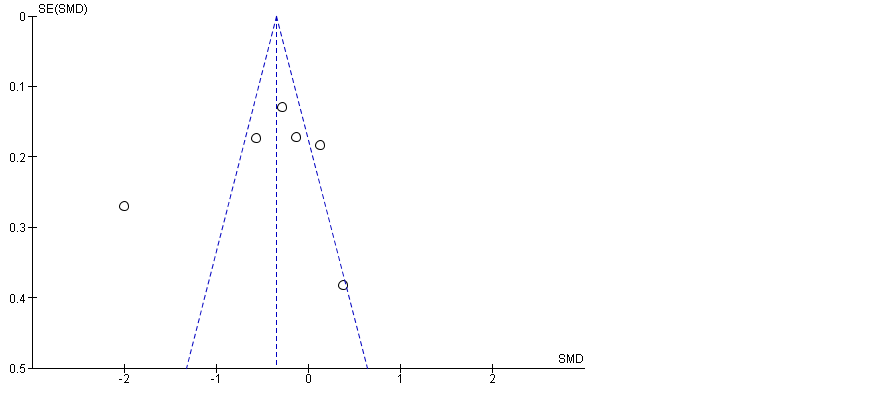


**Figure S.m. C.c.** Funnel plot: fatigue


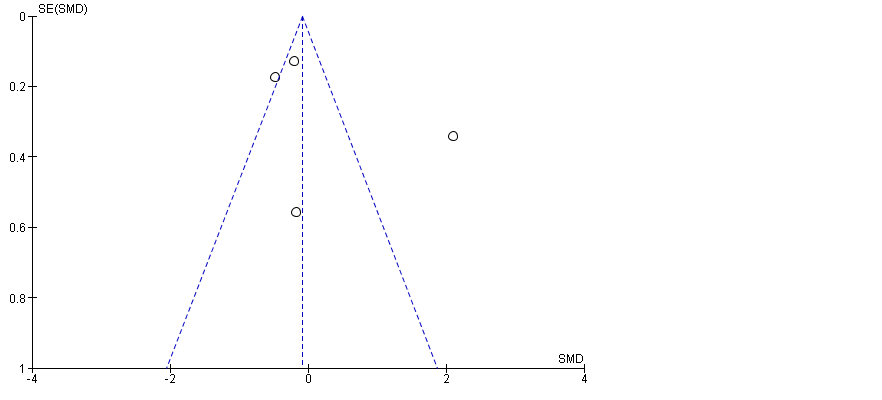

Supplement: Supplementary file 2 — Supplementary file2 (DOCX 56 KB) [file 520_2026_10331_MOESM2_ESM.docx]
